# Bodily disownership is associated with self-concept fragmentation

**DOI:** 10.1016/j.isci.2025.112805

**Published:** 2025-05-31

**Authors:** Pawel Tacikowski, H. Henrik Ehrsson

**Affiliations:** 1 Department of Neuroscience, Karolinska Institutet, Stockholm, Sweden; 2 Coimbra Institute for Biomedical Imaging and Translational Research, University of Coimbra, Coimbra, Portugal

**Keywords:** Behavioral neuroscience, Sensory neuroscience, Cognitive neuroscience

## Abstract

How does one’s sense of identity change when one feels disconnected from one’s own body? To explore this, we induced a perceptual illusion that diminished participants’ sense of ownership over their bodies using asynchronous visuotactile stimulation in sixty-six healthy individuals. Moreover, we asked them to rate how much certain personality traits described themselves. We found that reduced body ownership was linked to more dispersed self-description clusters. Control analyses confirmed that this main finding was not driven by unspecific semantic factors or asynchronous visuotactile stimulation alone. Thus, our results highlight the flexibility and interconnection between the bodily and conceptual aspects of the sense of self, which has important implications for dissociative experiences and overall mental health.

## Highlights

• Participants experienced a perceptual illusion of reduced body ownership
• Simultaneously, they rated how well various personality traits described them
• Reduced body ownership was associated with more dispersed self-description clusters
• The bodily and conceptual aspects of selfhood are flexibly interconnected

## Graphical abstract

**Figure:**
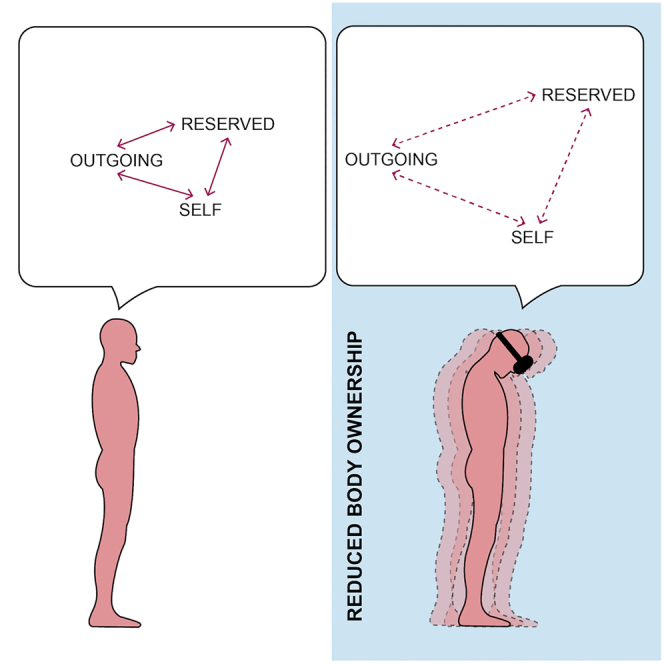

## Introduction

Our experience of the world is deeply rooted in our sense of self as the subject of experience. We perceive objects through our bodies and derive meanings from our past personal experiences.^1^^,^^2^ A key aspect of selfhood is the “bodily self,” which is the feeling of being separate from the external world and centered within a body that we perceive as our own.^3^^,^^4^^,^^5^^,^^6^ Another crucial component is the “self-concept,” comprising the various beliefs we hold about our identity and personality.^7^^,^^8^ Understanding the interaction between the bodily self and self-concept is a central challenge in modern psychology and neuroscience, particularly in relation to situations where the sense of self disintegrates.

In psychiatry and clinical psychology, dissociative experiences refer to disruptions or discontinuities in an individual’s normal sense of consciousness, memory, identity, or perception of the self and the environment.^9^^,^^10^ These experiences can vary in intensity and duration, ranging from mild feelings of detachment from reality to more severe, chronic conditions. While dissociative experiences can occur in many individuals without resulting in a disorder (e.g., during intense stress or trauma), when these experiences become chronic and severe, they are central features of a group of psychiatric conditions known as dissociative disorders.^9^^,^^10^ Alterations in bodily self-awareness are a common feature of dissociative experiences, often including a confusing and distressing loss of sense of ownership over one’s own body and detachment from the world and one’s sense of identity.^11^^,^^12^ These alterations frequently manifest as depersonalization and derealization, two dissociative symptoms that affect how individuals perceive their own body and the external world, respectively. However, dissociative experiences can also occur in other psychiatric conditions, such as post-traumatic stress disorder, borderline personality disorder, eating disorders, anxiety, depression, schizophrenia, and other conditions.^13^ Thus, revealing the neurocognitive mechanisms linking different components of the self and understanding how they are affected during dissociative experiences are crucial for improving mental health.

Experimental studies have demonstrated that manipulations of visuotactile or visuomotor synchrony create perceptual illusions where fake limbs^14^^,^^15^^,^^16^ or even entire artificial bodies seen from a first-person perspective^17^^,^^18^^,^^19^ become part of one’s bodily self. In turn, rendering visuotactile or visuomotor information asynchronous via augmented or mixed reality reduces the feeling that one’s own actual limbs^20^^,^^21^^,^^22^^,^^23^^,^^24^^,^^25^^,^^26^ or one’s whole body^27^^,^^28^ belong to oneself. Crucially, this experimentally induced “detachment” from the bodily self is especially pronounced among individuals who self-report frequent dissociative experiences in everyday life.^26^ Thus, evidence suggests that the brain’s mechanisms of multisensory integration play a key role in attributing ownership to one’s body,^3^^,^^4^^,^^6^^,^^29^ and a disruption of such integration is related to dissociative experiences. A crucial question is what happens to one’s sense of identity when one feels “detached” from one’s body?

Here, we take a new approach to previously published data^28^ to examine how a reduced sense of body ownership affects the structure of self-concept. Self-concept is likely organized around multiple aspects, such as ethnicity, gender, age, and social or professional roles.^30^^,^^31^ The structure of self-concept may be related, for example, to the number of aspects of self-concept or the consistency between them.^31^ Some recent studies,^32^^,^^33^^,^^34^ including our own,^28^ have extended this framework by analyzing the similarity (or dissimilarity—distance) between behavioral or neural responses to all pairs of trait adjectives rated by an individual. This approach can identify distinct clusters of self-descriptions and provide a fine-grained proxy for the structure of self-concept. For example, someone who perceives themselves as “outgoing” might also see themselves as “sociable” and “cheerful,” as these traits are typically associated with being outgoing but not with being “anxious” or “introverted.” The number and extent of such clusters represent the structure of self-concept, which is distinct from its content. Drawing on theories of embodied cognition,^35^^,^^36^ subjective experiences of depersonalization,^11^^,^^12^ and existing evidence highlighting the importance of the bodily self for episodic memory,^28^^,^^37^^,^^38^^,^^39^^,^^40^^,^^41^ we hypothesize that a reduced feeling of body ownership alters the structure of the self-concept, quantified as changes in the dissimilarity/distance between subjective ratings of one’s own personality traits.

## Results

**Figure 1:**
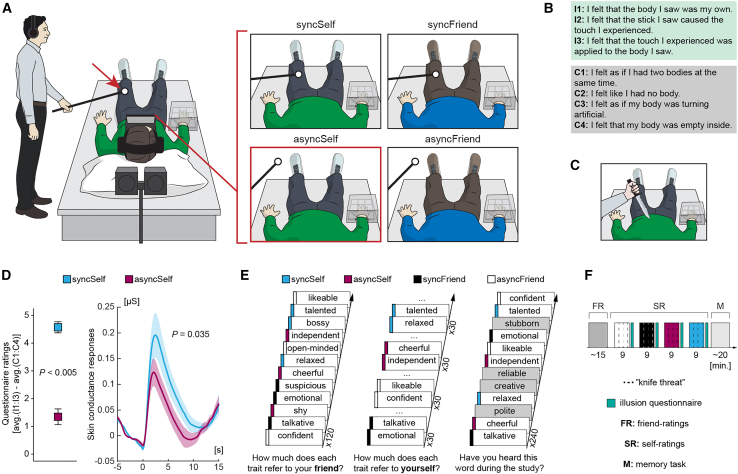
Methods (A) Experimental setup. Schematic representation of the setup and four experimental conditions for one participant in a dyad. The participants lay on beds with their heads tilted forward, wearing head-mounted displays (HMDs) connected to video cameras placed right behind and above their heads, providing a natural view of their own or their friend’s body. The touches felt by the participant and touches seen via the HMDs were matched in the synchronous (top) and delayed in the asynchronous conditions (bottom). (B) Illusion questionnaire. After each condition, the participants rated three illusion (I1:I3) and four control (C1:C4) statements on a 7-point scale (−3 “strongly disagree;” +3 “strongly agree”). (C) Knife threats. A genuine feeling of body ownership should be associated with increased physiological stress responses when this body is physically threatened. Conversely, genuine “detachment” from one’s own body should be related to decreased stress responses in that situation. To test this hypothesis, we simultaneously “attacked” both participants’ bodies with mock knives and measured skin conductance responses during these events. (D) Questionnaire and skin conductance results. Compared with the syncSelf condition, both methods showed significantly reduced responses in the asyncSelf condition, indicating a reduced feeling of body ownership during the latter.^28^ Plots show means ± SEM. (E) Friend rating, self-rating, and memory tasks. At the beginning of the study, the participants listened to 120 trait adjectives and rated how well each described their friend (1 “not at all;” 9 “very much”). The same traits were then randomly assigned to the four conditions, and, during each condition, the participants rated how well each trait described themselves. Finally, the participants listened to the same trait adjectives as before, now mixed with 120 new traits, with the task of determining whether each word had been presented earlier in the study or not. (F) Timeline. The condition order was randomized across participants. The color labels are the same as those in (E). Figure adapted from Tacikowski et al.,^28^ with permission.

We begin by summarizing key elements of our previously published study^28^ on which the current new analyses are based. It involved 66 healthy participants who were paired with a friend and took part simultaneously as 33 dyads. While lying on beds, participants viewed live recordings from cameras placed above either their own or their friend’s head using head-mounted displays (HMDs). Simultaneously, the experimenters applied strokes to the participants’ abdomens and upper legs using small balls attached to the end of a thin wooden stick (Figure 1A).

In the “synchronous-self” (syncSelf) condition, which served as a baseline, participants viewed their own body from a natural first-person perspective, and the touches they felt on their body (which they could not directly see) aligned with the touches they observed through the HMDs. Since the 3D visual image of their own body being stroked matched in space and time with the somatosensory experience of their real body, this condition simulated the normal default sense of body ownership, mirroring everyday experiences. In the “asynchronous-self” (asyncSelf) condition, which is most relevant to the present study, participants saw their own body from a first-person perspective while experiencing the 3-second visuotactile delay. This mode of asynchronous visuotactile stimulation reduces the sense of ownership over one’s body, creating a feeling of detachment from it, as if the body no longer feels like one’s own, even though participants can still visually recognize it as their own.^20^^,^^28^^,^^42^

The study also included two additional conditions, which are less central to the current study but provide valuable control conditions for unspecific full-body illusion and visuotactile asynchrony effects. In the “synchronous-friend” (syncFriend) condition, participants viewed their friend’s body from a first-person perspective, and the touches they felt on their (unseen) real body matched those they observed on their friend’s body through the HMDs. This created the perceptual illusion that their friend’s body was their own.^28^ Conversely, in the “asynchronous-friend” (asyncFriend) condition, the visuotactile stimulation was delayed by 3 seconds, eliminating the “friend-body-swap illusion” and serving as a well-matched control.

The subjective experience of body ownership in the four conditions was quantified using a questionnaire in which participants rated their experiences on a Likert scale (Figure 1B; STAR Methods). As reported in the original study,^28^ these data confirmed a significant (*p* < 0.05) reduction in subjective body ownership in the asyncSelf condition compared with the syncSelf condition (Figure 1D). In addition, skin conductance responses triggered by physical threats directed toward the body in view (Figure 1C) provided significant (*p* < 0.05) physiological evidence that, compared with the syncSelf condition, asynchronous visuotactile stimulation of the real body (asyncSelf) reduced body ownership (Figure 1D; STAR Methods).

Crucially, the participants also completed two personality-rating tasks (Figures 1E and 1F). The “friend-rating task,” conducted at the start of the experiment, required participants to rate how well 120 trait adjectives described their friend. In the subsequent “self-rating task,” participants rated how well the *same* traits applied to themselves (sets of 30 traits were randomly assigned to the syncSelf, asyncSelf, syncFriend, and asyncFriend conditions). Thus, the participants reflected upon their self-concept while feeling somewhat “detached” from their body during the asyncSelf condition, making this situation ideally suited to explore this study’s main research question of how reduced body ownership affects the structure of self-concept.

At the end of the study, the participants performed a memory task. Seated in front of computers, they listened to the same trait adjectives as before, now mixed with 120 new traits. Their objective was to identify whether each word had been presented earlier in the study (Figures 1E and 1F). Since the participants were not informed about this memory test in advance, incidental episodic recognition memory was assessed.^43^

**Figure 2:**
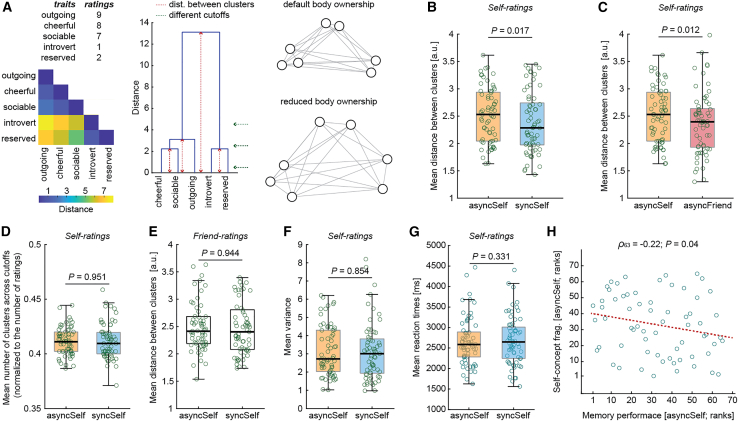
Analysis and results (A) Schematic illustration of self-concept as a relational representation (left). The distances between the ratings of each pair of traits can be plotted as a matrix. Traits rated similarly cluster together (small distances correspond to blue areas). This matrix can then be used as input for hierarchical clustering analysis (middle). We hypothesized that reduced ownership of one’s body would reorganize the structure of one’s self-concept, for example, by making the clusters more dispersed (right; nodes represent traits, and lines represent distances). (B and C) We found that the mean distance between clusters of self-ratings was significantly greater in the asyncSelf than in the syncSelf and asyncFriend conditions. (D) The asyncSelf and syncSelf conditions did not differ significantly in terms of the number of detected clusters. (E–G) Control analyses revealed that (1) the body-induced fragmentation of self-concept was specific to self-descriptions, as there were no analogous significant differences for friend ratings of traits that were later assigned to asyncSelf and syncSelf, and (2) this effect was not simply due to generally more unsystematic responses, as the differences in variance and reaction times were not significant. (H) Greater structural fragmentation of self-concept was related to decreased memory performance for traits rated during the asyncSelf condition. In all panels, the central marks of the boxplots indicate the medians. The bottom and top edges of the boxes indicate the 25th (Q1) and 75th (Q3) percentiles, respectively. The whiskers extend to the most extreme data points not considered outliers (1.5 × interquartile range above Q3 or below Q1).

For the main analysis, we calculated Euclidean distances between self-ratings for every pair of personality traits. In other words, we measured how differently each trait was rated compared with every other trait (e.g., with three traits, the complete list of pairs would be outgoing-cheerful, outgoing-sociable, and cheerful-sociable; Figure 2A). Traits rated similarly resulted in small distances (i.e., they belonged to the same cluster), whereas traits rated differently resulted in large distances (i.e., separate clusters). This analysis was conducted separately for each participant and condition. The resulting distance matrices were used as inputs for hierarchical clustering analysis, a classical method for detecting clusters based on pairwise distances between them. The distance between clusters provides an intuitive index of the degree of system fragmentation, which was the main focus of the present study. For alternative methods that should yield analogous results, please see previous work.^32^^,^^33^^,^^34^ We hypothesized that the organization of self-concept clusters would be specifically altered during the asyncSelf condition.

We found a significant interaction between the “synchrony” and “person” factors (*F*_1,64_ = 5.36; *p* = 0.024; repeated-measures ANOVA). The mean distance between self-description clusters was significantly greater in the asyncSelf than in the syncSelf condition (Figure 2B; t_64_ = 2.46; *p* = 0.017; paired t test; two-sided). The degree of this “self-concept fragmentation” was also greater in the asyncSelf than in the asyncFriend condition (Figure 2C; t_64_ = 2.59; *p* = 0.012; paired t test; two-sided), indicating that self-concept fragmentation was not driven by asynchronous visuotactile stimulation as such or by not experiencing ownership of the body in view; rather, it was specifically related to losing the feeling of ownership over one’s own body. The difference between the asyncSelf and syncFriend conditions was also marginally significant (t_64_ = 1.92; *p* = 0.059; paired t test; two-sided; see also Figures S2 and S3). These results suggest that reduced ownership of one’s own body is associated with a dispersed self-concept structure, and this effect seems specifically related to the perception of one’s own actual body.

To test whether there were generally more self-concept clusters in the asyncSelf condition, we again performed hierarchical clustering, but this time with a range of different cutoff values (see STAR Methods). For each cutoff, we checked how many clusters were detected. We found that the average number of clusters (across all cutoffs) did not differ significantly between the asyncSelf and other conditions (asyncSelf vs. syncSelf: Figures 2D and S1; t_64_ = 0.06; *p* = 0.951; BF_01_ = 7.34; asyncSelf vs. asyncFriend: t_64_ = 1.19; *p* = 0.238; BF_01_ = 3.74; asyncSelf vs. syncFriend: t_64_ = −0.12; *p* = 0.901; BF_01_ = 7.3; paired t tests; two-sided; BF_01_, Bayes factors supporting the null hypothesis). These results suggest that the loss-of-ownership-induced fragmentation of self-concept was specifically related to the distance between clusters of self-descriptions, not their number.

Next, we performed control analyses to exclude alternative explanations of the aforementioned findings. For example, one could argue that greater distances between clusters in the asyncSelf condition were due to generally weaker semantic links between trait adjectives from this condition. In other words, traits randomly assigned to the asyncSelf condition may have been less “semantically clustered” than traits assigned to other conditions. If that was the case, clusters of *friend ratings* provided by the same participants for exactly the same trait adjectives should also be more dispersed. To test this possibility, we applied separate hierarchical clustering to friend ratings of specific subsets of traits that were later assigned to the asyncSelf and syncSelf conditions. We found that the distance between clusters of those friend descriptions did not differ significantly between the two conditions (Figure 2E; t_64_ = 0.07; *p* = 0.944; paired t test; two-sided; BF_01_ = 7.34), suggesting that the main finding was not simply due to general semantic features of traits from the asyncSelf condition.

Another possibility we wanted to rule out is that the asynchronous visuotactile stimulation in the asyncSelf condition distracted the participants. If that had been the case, behavioral responses would have been slower and more unsystematic. However, we found no significant difference between the variance or reaction times of the responses in the asyncSelf versus syncSelf conditions (Figures 2F and 2G; variance: t_64_ = 0.18; *p* = 0.854; BF_01_ = 7.23; reaction times: t_64_ = −0.98; *p* = 0.331; BF_01_ = 4.65; paired t tests; two-sided; please note however that providing self-ratings was not a speeded task). Thus, self-concept fragmentation in the asyncSelf condition is unlikely to be due to general attentional distraction.

Finally, we conducted exploratory correlation analyses. While the relationship between self-concept fragmentation and reduced body ownership in the asyncSelf condition was not significant (questionnaire: ρ_63_ = −0.044; *p* = 0.728; BF_01_ = 9.66; skin conductance: ρ_63_ = −0.177; *p* = 0.16; BF_01_ = 3.84; Spearman correlations; two-sided; *n* = 65), we found a marginally significant correlation between the distance of self-concept clusters and memory accuracy in the asyncSelf condition (Figure 2H; *ρ*_63_ = −0.22; *p* = 0.04; Spearman correlation; one-sided; *n* = 65). Notably, we previously reported that reduced body ownership in the asyncSelf condition is associated with impaired memory performance.^28^ These results suggest that detachment from one’s bodily self may interfere with episodic memory, leading to self-concept disintegration. However, this interpretation should be approached with caution, as our mediation analysis (see STAR Methods) did not yield significant results. Alternatively, the study may have lacked sufficient statistical power to detect more complex mediations between bodily disownership, episodic memory, and self-concept fragmentation.

## Discussion

This research explored the relationship between self-concept and the bodily self. By creating an illusion in which participants felt “detached” from their physical body, we examined how this altered bodily experience affected their ratings of their personality traits. The results indicated that a diminished sense of ownership over one’s body was associated with a more dispersed self-concept. In the following, we discuss the implications of these findings for understanding the mechanisms underlying self-coherence and dissociative experiences.

Previous studies have demonstrated that experimentally altering the bodily self leads to distinct cognitive, emotional, and behavioral outcomes. For example, attitudes toward a racial group shift after embodying a member of that group^44^^,^^45^ and full-body ownership illusions can influence emotional responses such as social fear^46^ and body dissatisfaction.^47^ Similarly, the perception of one’s own body can modify the internal representation of one’s self-face,^48^^,^^49^^,^^50^ as well as one’s behavioral tendencies^51^ and implicit associations with the past self.^52^ In our previous work,^28^ we showed that, when participants experienced illusory ownership of a friend’s body, they began to rate their personality traits more similarly to how they had previously rated their friend’s traits, indicating that self-concept adapts to the bodily self to maintain a coherent sense of selfhood in a current situation. The present study expands on this by showing that, when the bodily self “disintegrates,” the structure of self-concept becomes more fragmented.

Arguably, in the critical asyncSelf condition, the spatial coherence of bodily sensations became less tight, and visual and somatosensory sensations became less perceptually bound than in the syncSelf condition, where people experienced their body much like in everyday life. This diminished sense of body ownership in the former condition likely led to a “loosening” of the relationships among self-concept aspects. In terms of neurocognitive mechanisms, we suggest that the brain encodes relational knowledge, such as self-concept, similar to how it represents spatial relationships in the physical world.^53^^,^^54^^,^^55^^,^^56^ In the theoretical framework of conceptual spaces pioneered by Peter Gärdefors, even abstract knowledge has a geometric or spatial structure that mirrors physical space. We argue that self-concept is organized analogously on the basis of continuous dimensions. Since the representation of physical space is based on the perception of the body and its environment, we suggest that there is a natural relationship between bodily space and self-concept space. If this is the case, we theorize that uncertainty about bodily boundaries, self-location, and multisensory spatial coherence of one’s body in the asyncSelf condition may trigger a corresponding uncertainty and reduced coherence of self-knowledge encoded in self-conceptual space. Such bodily uncertainty may also impair the ability to make inferences (“navigate”) within conceptual spaces for which self-concept is the reference point.^57^^,^^58^ Although little is known about the cognitive mechanisms or spatial transformations that may link the sense of body ownership, bodily perceptual space, and self-concept space, our findings provide preliminary experimental evidence pointing toward a fundamental connection. We know that changes in body ownership influence space perception^52^^,^^59^^,^^60^ and the sense of self-location.^27^^,^^61^^,^^62^^,^^63^ For example, when individuals experience the loss of ownership of a full body in illusion paradigms, this leads to greater uncertainty and a less clear sense of self-location.^64^ Thus, we theorize that such spatial effects also involve the structure of self-concept. At the neural level, this might occur through functional interactions between the multisensory fronto-parietal areas that represent perceptual aspects of the bodily self^15^^,^^18^; the medial prefrontal region involved in self-concept representation^65^^,^^66^; and the hippocampal-retrosplenial system related to spatial navigation, episodic memory, and translation between allocentric and egocentric mental perspectives.^37^^,^^64^^,^^67^^,^^68^^,^^69^^,^^70^^,^^71^^,^^72^ Future neuroscientific research should explore whether and how the multivariate representation of self-concept in the prefrontal cortex is “remapped” during bodily illusions and the precise relationship between illusory changes in specific spatial aspects of bodily representation and the corresponding remapping of self-concept space.

Our findings also offer new insights into the relationships among body ownership, self-concept, and memory. While previous research, including our own, has proposed that the loss of body ownership may influence memory encoding via hippocampal mechanisms,^37^^,^^72^ the current results suggest an additional downstream effect of this influence. We found that poorer episodic memory performance was associated with increased self-concept fragmentation. Although this correlation was only borderline significant, it may provide valuable insights into how body ownership influences higher cognitive functions—a relationship empirically demonstrated in numerous studies^73^—for which a mechanistic understanding has remained elusive. A coherent self-concept has been proposed to be critically linked to personal memories,^74^^,^^75^ and disruptions in self-concept, as seen in dissociative disorders, are often associated with memory impairments.^76^ However, the specific link between the structure of self-concept and episodic memory has been less explored. Our findings provide new evidence that, when the retrieval of episodic memories is disrupted through the loss of body ownership, it may loosen the structure of self-concept, which in essence corresponds to evaluating recalled personal experiences and mapping relationships between different traits. These insights highlight the complexity of this relationship and call for further experiments on how self-concept fragmentation, episodic memory, and reduced body ownership experience are related, particularly in clinical populations, such as those with dissociative disorders, where self-identity, memory, and bodily awareness are impacted.

As highlighted in the introduction, increasing evidence suggests that dissociative experiences arise from atypical multisensory integration of signals related to the body and the external world.^20^^,^^21^^,^^23^^,^^24^^,^^25^^,^^26^ Unlike prior studies, we focused on a diminished sense of ownership over the entire body, which more closely aligns with the phenomenology of dissociative experiences than the disownership of individual limbs.^12^ Importantly, we extended existing knowledge by examining the cognitive consequences of disrupting the bodily self—we discovered that this disruption dynamically reorganizes self-concept. This key finding resembles the identity uncertainty symptoms reported by individuals experiencing high levels of dissociation.^12^ The “full-body disownership illusion” is also associated with impaired episodic memory,^28^^,^^72^ another hallmark of dissociative experiences.^11^ Therefore, bodily illusions similar to the one used here present a promising experimental model to study dissociation and depersonalization conditions.

In conclusion, this study explored the intriguing question of how one’s sense of identity is affected by the feeling of “detachment” from one’s own body, similar to a dissociative experience. We found that only a few minutes of asynchronous visuotactile stimulation led to a diminished sense of ownership over one’s actual body, along with a structural reorganization of multiple subjective ratings of one’s personality traits. These findings highlight the remarkable flexibility of the bodily and conceptual aspects of the self and their close interconnection. Beyond its general relevance to all individuals as embodied beings, this malleability of selfhood has significant implications for mental health.

### Limitations of the study

One limitation is that the functional significance of a dispersed self-concept remains unclear. More distinct self-description clusters could indicate greater self-concept clarity, which is generally considered psychologically adaptive.^31^^,^^77^ However, it could also suggest less consistency and greater internal conflict.^78^ Our finding that self-concept fragmentation was associated with poorer memory performance aligns more with the latter interpretation, but future research should examine the functional relevance of structural self-concept changes. Another limitation is that this study focused solely on ratings of one’s own personality. Thus, it remains uncertain whether other types of evaluations, social or nonsocial, might also become more fragmented when individuals feel detached from their body. The absence of significant differences in mean variances (Figure 2F) and reaction times (Figure 2G) between the asyncSelf and syncSelf conditions argues against this general impairment interpretation. Nonetheless, future studies are needed to investigate this possibility conclusively. It is also important to note that, when we use descriptors such as “fragmented” or “dispersed” self-concept, we are specifically referring to greater distances between clusters of self-descriptions, as this was the primary focus of our analysis. Other studies may employ alternative measures to capture different aspects of self-concept structure, such as density, centrality, recurring patterns, and so on. A further potential limitation is that our findings are based on an experimental model of dissociative experiences in healthy individuals. Future research should explore whether self-concept fragmentation also occurs in dissociation that arises from trauma, stress, or psychiatric conditions. Additionally, examining individual differences and potential links with dissociative traits in the general population would be highly valuable.

## Resource availability

### Lead contact

Requests for further information and resources should be directed to the lead contact, Pawel Tacikowski (paweltacikowski@gmail.com).

### Materials availability

All trait stimuli are provided in our previous article.^28^

### Data and code availability

• Anonymized source data are available in the Data S1.
• Our analysis did not involve the development of custom functions or toolboxes with broader applicability.
• Any additional information related to this paper is available from the lead contact upon a reasonable request.

## Acknowledgments

We would like to thank all the participants, Marieke L. Weijs for assisting with data collection for the original study on which the current article is based, Mattias Karlen for the illustration of the setup, and Martti Mercurio for important technical support. This study was funded by the 10.13039/501100004359Swedish Research Council (Distinguished Professor Grant 2017-03135; H.H.E.), Torsten Söderbergs Stiftelse, Göran Gustafsons Stiftelse, 10.13039/501100000780European Commission (MSCA fellowship; 750955; P.T.), and the 10.13039/501100001871Foundation for Science and Technology grant (2022.03499.CEECIND; P.T.).

## Author contributions

P.T. and H.H.E. designed the study and wrote the manuscript. P.T. analyzed the data. All authors provided revisions and approved the final version of the manuscript for submission.

## Declaration of interests

The authors declare no competing interests.

## STAR★Methods

### Key resources table

**Table:** 

REAGENT or RESOURCE	SOURCE	IDENTIFIER
**Software and algorithms**		

MATLAB 2019b	MathWorks Inc.	https://mathworks.com/products/MATLAB.html
Adobe Illustrator 2020	Adobe	https://www.adobe.com/products/illustrator.htm

### Experimental model and study participant details

#### Participants

A total of 66 healthy volunteers participated in the study (42 females; 64 right-handed; mean age: 26 ± 5 years). The sample size was determined on the basis of similar studies.^52^^,^^79^ We recruited same-sex friends who had known each other for at least 6 months (average: 3.5 years). All participants had either normal or corrected-to-normal vision, normal hearing, and no history of neurological or psychiatric disorders. One participant was excluded for failing to follow instructions. Written informed consent was obtained from all participants, and the study was approved by the Swedish Ethical Review Authority.

### Method details

#### Procedure

Pairs of friends participated together. Friendship aspects were first assessed using the Inclusion of Other in the Self scale^80^ and the Network of Relationships Inventory: Behavioral Systems Version.^81^ The participants then practiced using a keypad without looking (see the ‘full-body illusion paradigm’ section). A cardboard box covered their hand and the keypad, and the task was to press a key corresponding to a number displayed on the screen (20 trials). All the participants completed this survey successfully. Next, they performed the friend-rating task on separate computers, followed by the self-rating task during the syncFriend, asyncFriend, syncSelf, and asyncSelf conditions, without seeing each other’s responses. Each condition started with 45 s of illusion induction, followed by spoken instructions and the self-rating task itself. During each condition, we simultaneously ‘threatened’ both participants with mock knives and measured skin conductance responses. After each 9-min condition, the participants removed their headsets and filled out an illusion questionnaire. The following memory task was again performed in front of the computer. We concluded the study with individual debriefings (‘What result do you think we expect in this study?’; ‘Have you used any special strategy in any of the tasks, and if so, what was it?’; ‘Do you have any other comments or feedback?’). No participant guessed the study’s hypothesis.

#### Full-body illusion paradigm

The participants lay on two beds, wearing Oculus Rift headsets (Melo Park, CA, USA). They were instructed to relax and minimize movement, with their heads tilted forward by approximately 45°. Each headset was connected to two digital cameras (Grasshopper3, FLIR, Ludwigsburg, Germany) positioned parallel to each other and approximately 7 cm apart behind and above the participants’ heads. During synchronous conditions, the visual feed was displayed with minimal intrinsic delay (<100 ms), whereas a 3-s delay was introduced for asynchronous conditions. The participants received the same number of touches (∼13 per minute) on their upper legs and abdomen in each condition. The touches were applied using white Styrofoam balls (10 cm diameter) attached to thin wooden rods. The order of touches was predefined and pseudorandom, ensuring that no more than two consecutive touches to the same body part were applied. Each stroke lasted 1 s, with intervals of 2, 3, or 4 s between strokes, covering approximately 25 cm of the body. The experimenters practiced synchronizing their touches beforehand and followed audio cues played through their headphones during the study to maintain consistency in the onset, duration, and location of the strokes. The stroking was paused after 10, 20, and 30 self-ratings. During these pauses, both participants were simultaneously ‘attacked’ with mock knives. The participants’ right hands were covered with cardboard boxes to eliminate visual feedback from finger movements during self-ratings.

#### Illusion questionnaire

After each condition, the participants completed a questionnaire to measure the strength of the full-body illusion. Illusion and control statements (Figure 1B) were adapted from Petkova and Ehrsson,^17^ with participants rating agreement on a scale from −3 (‘strongly disagree’) to +3 (‘strongly agree’). The illusion statements focused on body ownership (I1) and referral of touch (I2, I3), the core aspects of the sense of body ownership.^3^^,^^29^ The control items (C1:C4) assessed suggestibility and task compliance. An additional statement, L1, explored changes in self-location during the friend-body swap condition. The order of statements was C1, I1, C2, I2, C3, C4, I3, and L1.

#### Skin conductance responses

Data were recorded using the Biopac System (MP150, Goleta, CA, USA) at a sampling rate of 100 Hz and processed with AcqKnowledge software (Version 3.9.1.6, Biopac). Two electrodes, applied with electrode paste, were placed on the participants’ left index and middle fingers (distal phalanges). Knife threats involved a stabbing motion that stopped just above the abdomen, each lasting approximately 2 s (Figure 1C). To prevent extensive emotional stress, the participants were shown mock knives before the study, in line with good ethical practice. Three threats occurred in each condition. The timings of these events were marked manually.

#### Friend- and self-reference tasks

Trait adjectives were selected from,^82^ focusing on items understandable to non-native English speakers and those showing the highest variability in a pilot study (*n* = 10). A complete list of used traits is provided in our previous article.^28^ Presentation software (version 16.4, Neurobehavioral Systems, Inc., Berkeley, CA, USA) was used to present all the stimuli and record the responses. The participants heard the instructions and items through headphones and responded by pressing a key on a numeric keypad, rating how much the trait referred to their friend or themselves (1 = ‘not at all’; 9 = ‘very much’). Each trait was preceded by a 200 ms beep sound, and traits were presented for an average duration of 0.8 ± 0.1 s. The participants had up to 6 s to provide their ratings, with intertrial intervals of 1, 1.5, or 2 s. They rated the same 120 trait adjectives in both the friend- and self-reference tasks (30 randomly selected traits were assigned to the syncSelf, syncFriend, asyncSelf, and asyncSelf conditions, respectively). Thus, each condition contained a unique list of traits that the participants rated in relation to the self. We reasoned that repeatedly asking a person, for example, ‘How talkative are you?’ would have been confusing and suggestive—the participants might have wondered, ‘Why do they keep asking me the same question?’, ‘Should I change my response?’, ‘Are they testing if I remember what I said earlier?’, etc.

#### Memory task

A total of 120 ‘old’ items (from the friend- and self-rating tasks) were randomly mixed with 120 ‘new’ trait adjectives. The participants used the left and right mouse buttons to indicate whether they had heard each word earlier in the experiment, with key assignments counterbalanced between participants. The average stimulus duration was 0.7 ± 0.1 s, and participants had a maximum of 2.5 s to respond. After each response, feedback was provided (‘correct,’ ‘incorrect,’ or ‘too long’). The interval between trials varied, lasting 1, 1.5, or 2 s. We did not inform the participants about this memory test in advance; thus, we measured incidental episodic recognition memory.^43^

#### Analysis of illusion questionnaires

We computed ‘illusion scores’ by calculating the differences between the average ratings of illusion (I1:I3) and control (C1:C4) statements for each participant across the different conditions. The data were analyzed with the linear mixed model specified as ‘score ∼1 + condition +1|id,’ where the ‘condition’ refers to either asyncSelf or syncSelf, and ‘1|id’ represents the random intercept. Additionally, ratings for individual statements were assessed using pairwise Wilcoxon signed-rank tests (two-sided). Further details regarding the illusion questionnaire results can be found in our previous article.^28^

#### Analysis of skin conductance responses

The amplitude of each response was determined by calculating the difference between the maximum and minimum conductance values during the 0 to 6 s period following each knife threat. Amplitude values were square-root-transformed^83^ and analyzed using a linear mixed model defined as ‘response ∼1 + condition + repetition +1|id’. Here, the fixed effect of repetition, which ranged from 1 to 12, indicated each knife threat’s order throughout the experiment. Using a transformed repetition number (1/n) significantly improved the model fit to the data (χ^2^_1_ = 58.6; *p* < 0.001). For descriptive purposes, we plotted skin conductance responses as time courses (Figure 2D) via the following steps: (i) extracting segments from −10 s to +20 s around each knife threat; (ii) manually selecting the response onset; (iii) time-locking the signal to that onset; (iv) removing any linear trend using MATLAB’s ‘detrend’ function; (v) applying baseline correction (i.e., subtracting the mean value from the −5 s–0 s window; and (vi) averaging all trials from a given condition. Further details regarding the results of the skin conductance data can be found in our previous article.^28^

#### Analysis of friend and self-ratings

The number of personality traits actually rated was high (minimum of 20 traits per condition). These ratings exhibited the desired variability—in 99.7% of the condition-specific datasets, participants used five or more distinct rating values. Since some traits (e.g., aggressive) are generally rated lower than others (e.g., nice), a linear mixed model was applied with a random intercept for the trait type (rating ∼1|trait). Residuals from this model were then used to calculate distances between ratings (see the ‘clustering analysis’ section). This adjustment established a baseline for each trait and accounted for the fact that different conditions contained different traits (see the ‘friend- and self-reference tasks’ section), making the remaining variability more relevant to the present study. The model was estimated using friend- and self-ratings from all conditions combined, thus the between-session and between-condition comparisons were not biased by this pre-processing step.

#### Analysis of memory data

Only traits that were actually rated with a button press during the friend- and self-rating tasks were included in the memory analysis (7593 out of 7800), ensuring that the traits had been noticed during encoding. All ‘new’ traits (7800) were included. For each participant in each condition, we calculated d-prime values.^84^ At the group level, d-primes were significantly above chance level (t_64_ = 35.03; *p* < 0.005; one-sided), indicating high overall discrimination between old and new words. D-primes from each condition were mean-centered per participant to adjust for individual memory capacity differences.

#### Clustering analysis

For each participant and condition, we computed Euclidean distances between ratings of each trait pair. The resulting distance matrices were used in the hierarchical clustering analysis, which was performed with MATLAB’s ‘linkage’ function (method: ‘average’). This function takes a single input—the distance matrix—and outputs distances between all detected clusters. For the group-level analysis, we used the mean distance between all clusters calculated per participant and per condition for either self-ratings or friend ratings. To test whether there were generally more self-concept clusters in the asyncSelf condition, we performed hierarchical clustering across a range of different cutoff values. Specifically, clusters were considered distinct if the distance between them exceeded a specified threshold, implemented using MATLAB’s ‘cluster’ function with cutoff values ranging from 0.1 to 1.5 in increments of 0.1. The logic of this procedure is illustrated in Figure 2A (middle panel), where the number of detected clusters varies depending on the cutoff value (green arrows). As shown in the Figure S1, the number of clusters saturates at the lowest and highest cutoffs, demonstrating that our selected range is sufficiently wide. We then calculated the average number of clusters across all cutoff values for each participant in each condition. Please note that there is no universally optimal cutoff value for hierarchical clustering, as the appropriate choice depends on the dataset and the specific objectives of the analysis.^85^ Given that our study included multiple datasets per participant and per condition, we chose not to pursue the identification of a single optimal cutoff value. Instead, our approach—averaging the cluster distances across multiple cutoffs—uses the full structure of the dendrogram^85^ and appears well-suited to address our research question of whether the asyncSelf condition was *generally* associated with a greater number of clusters. Notably, the number of clusters could be affected by the total number of ratings—more items means more possible clusters. To correct for this, we calculated proportions and used them in the subsequent analyses (Figure 2D; mean number of clusters in a given condition divided by the total number of ratings in that condition).

#### Correlation analyses

The index of reduced body ownership was calculated as the difference between the syncSelf and asyncSelf conditions. This was done for both the questionnaire and skin conductance responses. For questionnaires, we specifically focused on the ratings of the I1 ‘ownership statement’ (Figure 1B). For skin conductance, we first extracted residuals from the model ‘response ∼1 + repetition’ and then computed the difference between average responses in syncSelf minus asyncSelf for each participant. This approach reduced the confounding variance due to habituation.^83^ The degree of self-concept fragmentation was calculated as the difference between asyncSelf and syncSelf for distances between self-rating clusters. Finally, we correlated memory performance (d-primes) for items presented during the asyncSelf condition and the degree of self-concept fragmentation in asyncSelf (mean distance between clusters; z-scored per participant).

#### Mediation analysis

We used the ‘M3’ MATLAB toolbox^86^ to test a mediation model in which memory performance mediates the effect of body disownership on self-concept fragmentation. This model consisted of a direct path from bodily disownership to self-concept fragmentation and two indirect paths: (i) from bodily disownership to memory performance and (ii) from memory performance to self-concept fragmentation. The variables were defined as follows (as previously explained): disownership was calculated as the difference in ratings of the I1 ownership statement between the syncSelf and asyncSelf conditions; memory performance was measured as d-primes for items presented during the asyncSelf condition; and self-concept fragmentation was quantified as the mean distance between clusters of self-ratings during the asyncSelf condition (z-scored). The mediation effect was not statistically significant (z = −0.34; *p* = 0.738).

### Quantification and statistical analysis

All analyses were performed in MATLAB version R2019b. To compute Bayes factors, we used the ‘bayesFactor’ package (Cauchy prior; 0.7071; https://github.com/klabhub/bayesFactor).

## Supplemental information

Document S1. Figures S1–S3

Data S1. Anonymized source data (A) Source data for Figure 2B. (B) Source data for Figure 2C. (C) Source data for Figure 2D. (D) Source data for Figure 2E. (E) Source data for Figure 2F. (F) Source data for Figure 2G. (G) Source data for Figure 2H.
