# Supplementary material for: Bodily disownership is associated with self-concept fragmentation
Source: iScience. 2025 May 31;28(7):112805. doi: 10.1016/j.isci.2025.112805 (PMC12205829; doi:10.1016/j.isci.2025.112805)
Supplement: Document S1. Figures S1–S3 [file mmc1.pdf]

iScience, Volume *28*

Supplemental information

Bodily disownership is associated with self-concept fragmentation

Pawel Tacikowski and H. Henrik Ehrsson

This document includes the following:

Supplementary Fig. S1

Supplementary Fig. S2

Supplementary Fig. S3

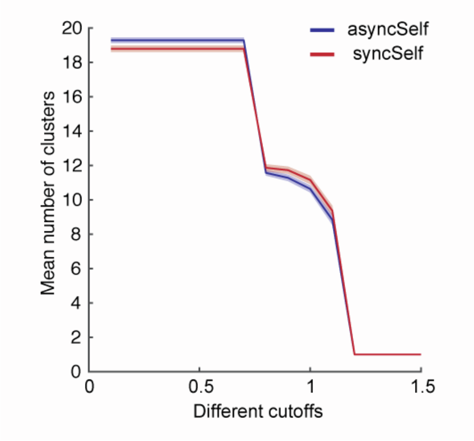

Fig. S1. The number of clusters across all cutoffs, Related to STAR Methods (*Clustering analysis*). As expected, generally less clusters were detected with higher cutoffs. There were no prominent differences between the asyncSelf and syncSelf conditions across all cutoffs. The plot shows means ± SEM. Data is self-ratings.

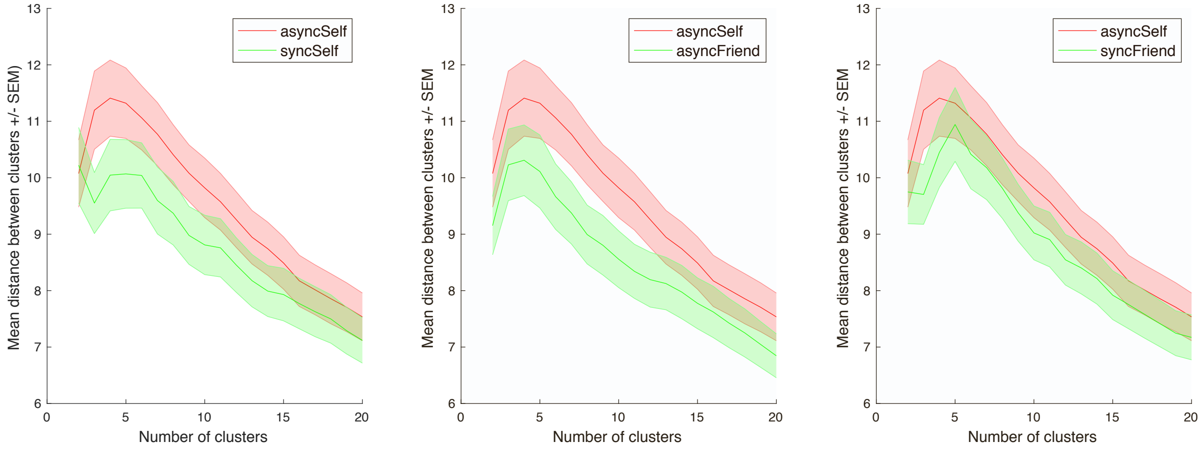

Fig. S2. Between cluster distances, Related to Fig. 2C and 2D. We performed k-medoids clustering on data from each condition and each participant separately ('kmedoids' MATLAB function; the number of clusters ranging from 2 to 20). To analyze between-cluster distances, we calculated the sum of medoid-to-medoid distances between clusters. Visual inspection suggests that (i) as the number of clusters increased, the distances between clusters decreased, which is expected, and (ii) distances between clusters in the asyncSelf condition were generally larger than in the other conditions.

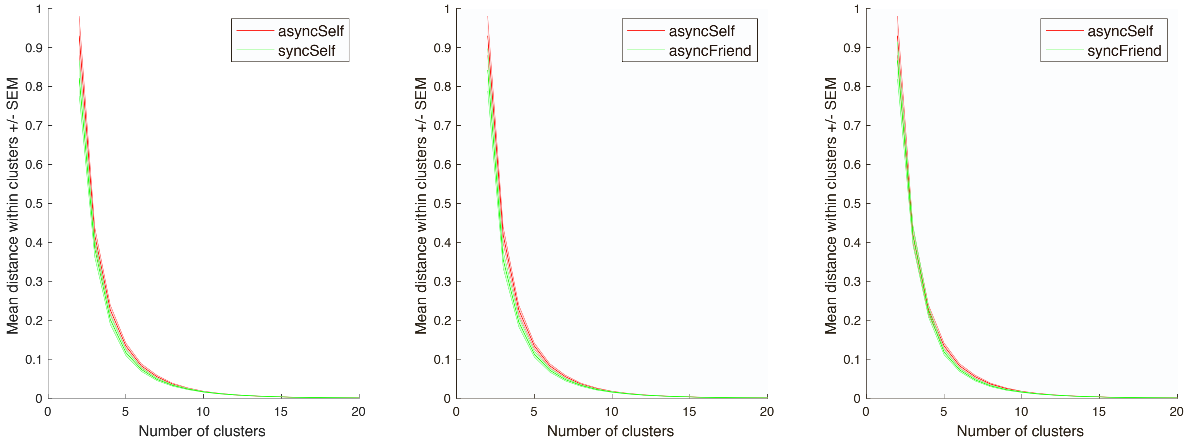

Fig. S3. Within-cluster distances, Related to Fig. 2C and 2D. We performed the same analysis as described earlier (Fig. S2), but we focused on the sums of point-to-medoid distances. Visual inspection of the plots suggests that the differences between conditions are minimal.
